# Applications of leech therapy in medicine: a systematic review

**DOI:** 10.3389/fmed.2024.1417041

**Published:** 2024-09-16

**Authors:** Mahsa Hosseini, Ali Jadidi, Mohammad Moein Derakhshan Barjoei, Mehdi Salehi

**Affiliations:** ^1^Students Research Committee, Arak University of Medical Sciences, Arak, Iran; ^2^Department of Nursing, School of Nursing, Arak University of Medical Sciences, Arak, Iran; ^3^Virtual School of Medical Education and Management, Shahid Beheshti University of Medical Sciences, Tehran, Iran; ^4^Student Research Committee, Shahid Sadoughi University of Medical Sciences, Yazd, Iran; ^5^Traditional and Complementary Medicine Research Center, Department of Traditional Medicine, School of Medicine, Arak University of Medical Sciences, Arak, Iran

**Keywords:** leech therapy, clinical trial, systematic review, hirudotherapy, complementary medicine

## Abstract

**Background:**

Leech therapy (LT) is one of the most widely used treatment methods in traditional medicine. The present study aimed to systematically review clinical trials regarding the effects of LT on the prevention and healing of different diseases.

**Methods:**

To identify all relevant published studies, we conducted a comprehensive search of PubMed, Scopus, and Web of Science databases without any temporal or geographical constraints until April 2023. To categorize the articles, five stages were considered. The PRISMA checklist and Cochran’s bias analysis tool were used.

**Results:**

In total, 12 trials that met the inclusion criteria were studied. The results of the studies showed that LT has had successful outcomes in treating different conditions. These included hormonal and metabolic complications, cardiovascular problems, and inflammatory-based diseases.

**Conclusion:**

“Leech therapy” is a traditional medical treatment used successfully to control and treat various conditions. Although this method can have complications, it is possible to benefit from this low-cost and low-complication treatment by taking preventive measures.

## 1 Introduction

Leeches, characterized by their bloodthirsty traits, have been used in diverse traditional medical practices due to the therapeutic attributes of their saliva. This therapeutic approach is called LT (1–4). The leech–host interaction represents a direct connection that occurs when an external parasite, such as a leech, bites its host and begins to extract blood (5). In LT, each leech ingests 5–15 mL of blood, and typically, practitioners use one or two leeches for a duration of 20–120 min (6, 7). During feeding, leech saliva releases a rich array of bioactive compounds into the host’s bloodstream. These compounds exhibit diverse functions, including platelet inhibition, anticoagulation, anti-inflammatory effects, thrombin regulation, vasodilation, analgesia, and antimicrobial properties. Moreover, it contains some enzymes, such as eglins, bdellins, hirudin, destabilase, and calin, which are beneficial for skin wound healing (8, 9).

LT, utilizing the bioactive components present in leech saliva, has been used in the management of diverse acute and chronic conditions. These include preventing venous congestion after trauma and surgery in post-phlebitic syndrome, cardiovascular diseases, deep vein thrombosis, acute and chronic otitis, complications of diabetes mellitus, tinnitus, and osteoarthritis pain (10, 11). Another study indicated LT could be effective in glaucoma, arthritis, abscesses, myasthenia gravis, some venous disorders, and thrombosis (8). In Iranian traditional medicine (ITM), LT is used for local and skin problems, migraines, and chronic headaches (1, 2).

Among all of these, leech bites rarely cause some death and have dangerous potential in some cases. Other complications of LT are allergy, prolonged infection, and bleeding (5). Consequently, it is essential to assess and juxtapose the advantages of LTLT with its drawbacks. In this current review, we systematically summarize the therapeutic efficacy of LT for clinical applications.

## 2 Method

The current study was conducted according to the Prisma guidelines (12), and it aims to examine the applications and side effects of LT. The search was conducted without regard to place or time until 26 April 2023.

### 2.1 Search strategy

Initially, relevant keywords from Mesh and similar review articles were extracted. Then the search strategy was devised by the researcher (MH), and it received review and approval from the researcher (AJ). This study evaluated three databases: PubMed, Scopus, and Web of Science (Table 1).

**Table 1:** Search strategy.

Databases	Search queries	Result	Date
PubMed	(((“Leeches”[Mesh]) OR “Hirudo medicinalis”[Mesh]) OR “Leeching”[Mesh]) OR (((leech[Title/Abstract]) OR (“Hirudo medicinalis”[Title/Abstract])) OR (“leech therapy”[Title/Abstract]))AND (((((((“randomized controlled trial”[Title/Abstract]) OR (“randomized controlled trial”[Title/Abstract])) OR (“randomized”[Title/Abstract])) OR (“randomized”[Title/Abstract])) OR (“controlled clinical trial”[Title/Abstract])) OR (“RCT”[Title/Abstract])))	33	26 April
WOS	TS = (“Leeching “OR “Leech Therapy “OR “Leeches” OR “Hirudo medicinalis”) AND TS = (“randomized controlled trial” OR “randomized controlled trial” OR “randomized” OR “randomized” OR “controlled clinical trial” OR “RCT”)	33	26 April
SCOPUS	TITLE-ABS (leeching OR “Leech Therapy” OR “Leeches” OR “Hirudo medicinalis”) AND TITLE-ABS (“randomized controlled trial” OR “randomized controlled trial” OR “randomized” OR “randomized” OR “controlled clinical trial” OR “RCT”)	44	26 April

### 2.2 Screening

All results were entered into Endnote version X9 software. In the screening articles, five stages were considered: 1. eliminating duplicates, 2. reviewing the article titles and abstracts to verify that they are appropriate to the study’s aims, 3. reviewing the full text of the articles, 4. analyzing the inclusion and exclusion criteria, and 5. examining the references of the selected articles to identify relevant studies that can be integrated into the research. Furthermore, during all stages of the research, each potentially problematic phase of the study underwent assessment by an independent investigator.

### 2.3 Inclusion and exclusion criteria

Inclusion Criteria:

We focused on randomized clinical trials (RCTs) as our primary study design. Only studies with available full-text content in English were considered. Participants meeting these criteria were eligible for inclusion.

Exclusion Criteria.

We excluded other types of studies beyond RCTs. Studies lacking complete English text were also excluded. Additionally, we applied criteria to exclude studies with a serious risk of bias.

### 2.4 Data extraction

Extracted data based on populations/interventions (leeches)/controls/outcomes (PICO) outline include the first author’s name, year of publication, sampling site, sample size, type of disease (population), mean age or age of participants, intervention (the method of duration, location of leeches, etc.), application, side effects, and consequences.

### 2.5 Risk of bias

The risk of bias in randomized articles was assessed using Cochran's bias analysis tool (RoB 2) (13). Based on this tool, bias in seven areas: 1. difficult to understand 2. Selecting participants. 3. Intervention classification. 4. Deviant learning and interventions. 5. Forgot information about the study and participants. 6. Assess the consequences. 7. A selection of reported outcomes is considered. This checklist, to provide an accurate assessment of bias in each of the seven areas, is classified into Low/High/Some concerns (Table 2).

**Table 2:** Risk of bias in randomized articles using Cochran’s bias analysis tool.

Author and year	Bias due to Confounding	Bias in the selection of participants into the study	Bias in the classification of interventions	Bias due to deviations from intended intervention	Bias due to missing data	Bias in the measurement of outcomes	Bias in the selection of the reported result	the overall risk of bias
Talebi et al. (21)	Low	Low	Low	Some concerns	Low	Low	Low	Low
Alemi et al. (2)	Low	Low	Low	Some concerns	Low	Low	Low	Low
Khodaverdian et al. (36)	Low	Low	Some concerns	Some concerns	Low	Low	Low	Low
Hohmann et al. (25)	Low	Low	Some concerns	Some concerns	Low	Low	Low	Low
Isik et al. (17)	Some concerns	Low	Low	Some concerns	Low	Low	Low	Low
Shiffa et al. (19)	Low	Low	Low	Some concerns	Low	Low	Low	Low
Stange et al. (20)	Low	Low	Low	Some concerns	Low	Low	Low	Low
Bäcker et al. (15)	Low	Low	Low	Some concerns	Low	Low	Low	Low
Nigar et al. (24)	Low	Low	Low	Some concerns	Low	Low	Low	Low
Zaidi et al. (23)	Low	Low	Low	Some concerns	Low	Low	Low	Low
Michalsen et al. (19)	Some concerns	Low	Low	Some concerns	Low	Low	Low	Low
Andereya et al. (15)	Some concerns	Low	Some concerns	Some concerns	Low	Low	Low	Some concerns
Michalsen et al. (18)	Low	Low	Low	Some concerns	Low	Low	Low	Low

Given the diversity of outcomes and examined variables across the studies, conducting a meta-analysis was not feasible in our research. Consequently, we performed a qualitative analysis of all findings. Furthermore, two researchers oversee the stages of article selection, data extraction, and quality assessment. In cases of disagreement, a third author is consulted.

## 3 Result

### 3.1 Quantitative results

**Figure 1:**
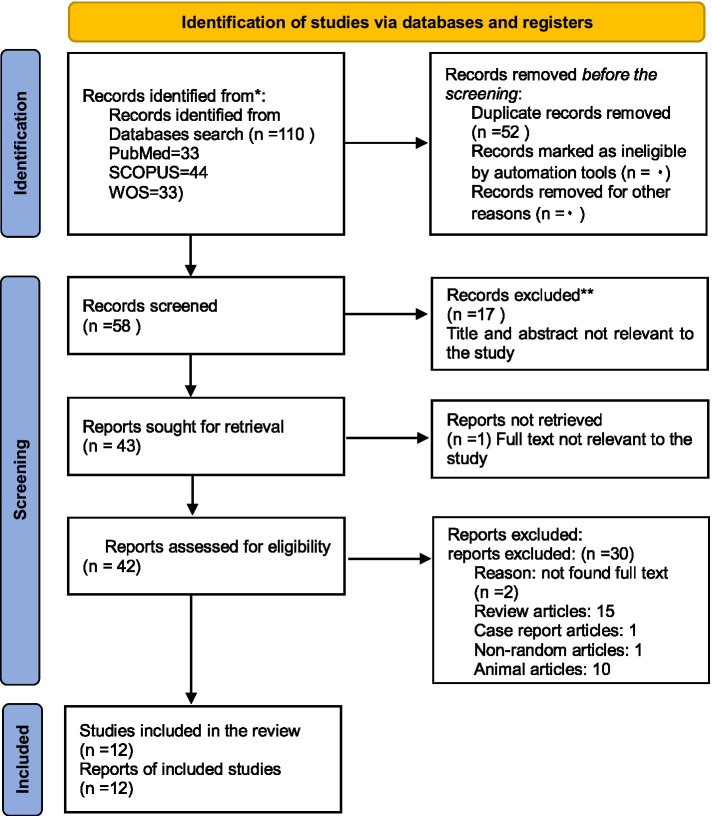
PRISMA checklist for article selection.

The purpose of the current study is to provide a comprehensive review of the benefits and drawbacks of LT. Finally, 12 articles relevant to the study’s objectives and meeting inclusion and exclusion criteria were selected (Figure 1). According to Cochran’s bias analysis tool, although there are some concerns about the quality of evidence in some items, except for one study, the rest of them were evaluated at a low level in terms of the overall risk of bias. Therefore, most of the studies used were of acceptable quality (Table 2). All studies were released between 2003 and 2022. Additionally, 723 individuals from both the control and intervention groups took part in these trials. The participants receiving LT and those with epicondylitis had an average age of 47.9 +/− 9.5 years (14). Furthermore, 68 + 10 was the highest average age among patients with knee arthritis (15). In the current research, men’s involvement rates ranged from less than 50% in 9 studies to 65% in one study to undefinable in two studies. These studies have looked at the following effects of LT on various medical conditions: eight articles on knee osteoarthritis (15–22), one on epicondylitis (14), four on varicose veins (23), one on chronic back pain (24), and one on type 2 diabetics with neurogenic diseases (2) have been conducted (Table 3).

**Table 3:** Summary of reviewed articles.

Author	Age (mean ± SD) in leech	Male (%) in leech	Assessment scales	Outcome	Population
Marcus Bäcker	47.9 ± 9.5	13/20: 65%	VAS/DASH/Short Form-36	Pain/disability/physical quality of life (Short Form-36), and grip strength.	Epicondylitis
Farshad Alemi	55.25 ± 9.61 (age range: 37 to 72 years)	6/20:30%	(VAS), Neuropathy Symptom Score (NSS), Neuropathy Disability Score (NDS), Nerve Conduction Velocity (NCV), and Electromyography (EMG)	(VAS), (NSS), (NDS), (NCV), (EMG)	Type II diabetes with lower limb diabetic neuropathy
Seyed Saman Talebi	61.44 ± 8.97	4% in all	KOOS questionnaires (five areas: pain, symptoms, performance in daily activities, performance in sports and recreational activities, and quality of life-related to knee function)	Short-and long-term outcomes of a knee injury	Knee osteoarthritis
Stefan Andereya	68 ± 10	44%	KOOS/ WOMAC/(VAS) for pain	KOOS/ WOMAC/(VAS) for pain	Osteoarthritis of the knee
M. Isik	59.6 ± 8.8	4%	(VAS)/WOMAC	Pain scores/WesternOntario and McMaster University Osteoarthritis Index/Secondary outcome changes in e WOMAC scores	Knee osteoarthritis
Zar Nigar	NA	68% in all	NA	Pain, discomfort in the leg, limb circumference in the leg, ankle, foot color Hemoglobin percentage: test Wade Assessment: Color Doppler Sono	Varicose veins
Christoph-Daniel Hohmann	59.29 ± 6.99	NA	(VAS)//(Roland–Morris Disability Questionnaire, Hannover Functional Ability Questionnaire)//Short-Form Health Questionnaire [SF-36]//pain perception scale = Schmerzempfindungsskala [SES]//Center for Epidemiological Studies Depression Scale [CES-D//questionnaire diary]	Average back pain intensity/functional impairment/quality of life/pain perception/depressive/analgesic consumption	Chronic low back pain
SM Abbas Zaidi	59.90 ± 2.79	45	Womac//all other Womac subscores eight-meter walk test	Pain score/active range of motion (knee flexion)	Knee osteoarthritis
Andreas Michalsen	62.5 ± 10.2	37	Subscores of the Western Ontario and McMaster Universities Osteoarthritis Index and physical sum score of the Medical Outcomes Study 36-Item Short-Form Health Survey with group comparison	Pain, function, and stiffness, quality of life	Osteoarthritis of the knee
Andreas Michalsen	64.1 ± 6.4	0%	VAS//(DASH-questionnaire)//(QoL, SF-36)	Pain/functional disability, quality of life, and grip strength	Osteoarthritis of the knee. Osteoarthritis of the first carpometacarpal joint (thumb saddle joint).
Mohamed Shiffa	51.53 ± 1.658	33	(VAS),KOOS.assess clinical efficacy.	/Pain/Knee injury/range of motion, 15-m walking time, and knee circumference were used to assess clinical efficacy.	Knee osteoarthritis
Rainer Stange	68.3 ± 10.2all	All 21%	Change in Lequesne’s combined index for pain and function and change (L.I.) and overall assessment of complaints by visual analog scale (VAS).	pain and function/complaints	Osteoarthritis of the knee

### 3.2 Intervention

#### 3.2.1 Several leeches

Various investigations have used diverse methodologies to quantify the leeches utilized in LT. Each treatment session typically involves a minimum of two and a maximum of eight leeches. Notably, some studies reported leech quantities using intervals rather than specific numerical values. Specifically, six studies recommend the use of five leeches per treatment session, while four studies advocate for four leeches. Additionally, three trials suggest using either two or three leeches during therapy sessions (2, 14, 18, 24).

#### 3.2.2 Duration and method of separation

In the present study, LT is effective for different durations of time, with a minimum duration of 30 min (21) and a maximum duration of 70 min (17, 19). However, the duration of LT did not show any correlation with the outcomes of the treatment.

Three studies (14, 15, 20) indicate the spontaneous removal of leeches from the treatment site, although another study mentions separation with NACL and the other study did not address this issue (21).

#### 3.2.3 Leech treatment places

The position of the leech is given variously according to the purpose of the study. Radial insertion of the extensor muscles of the wrist (mainly the extensor carpi radialis) to affect epicondylitis (14) and placement of leeches on the back of both feet (tangential from the base of the second toe to the lateral malleolus) to affect diabetic neuropathy in knee arthritis (2). The placement of leeches near the patella, on each side in the medial and lateral joint line of the knee, while the knee is comfortably in extension, intending to affect knee arthrosis (15) The placement of leeches 3–15 cm from the spine at the level of the L1-S3 vertebrae to reduce back pain (24), the placement of leeches with an examination of the soft tissue area of the joint around the big toe, to affect knee arthritis, focusing on the area that is most painful to the touch (18). Above and below the patella, as well as in the inner and lateral parts toward the joint cavity of the knee affect knee arthritis (20). Soft tissue around the joint on the inner side of the knee (mostly in the painful areas during the examination) and the lateral side of the knee joint (16), points around the knee joint (22). Soft tissue around the injured knee joint, preferably to the painful points during examination and palpation (17), placing two leeches, one on the inner side and the other on the side of the knee joint (19) to affect knee arthritis.

### 3.3 Harmful effects

None of the studies reviewed reported serious complications from LT. The two main complications reported in most studies are mild to moderate itching (14–18, 22, 24), mild bleeding (14, 15), and other complications such as decreased systolic blood pressure (15–20 mmHg), confusion for several minutes (14), extremity edema (2), local irritation (15), skin erythema (16, 18), decreased hemoglobin and hematocrit levels, and significantly increased PT, INR, and ESR values (16), bleeding lasting up to 24 h without anemia (24), and increased pain in the Hill therapy group (1 case) (24), and one case of exacerbation of arthritis (the authors said this was probably due to increased pressure on the knee and was unrelated to the study) (20).

### 3.4 Positive effects

According to the studies conducted, LT has positive effects such as reducing pain (2, 14–16, 18–20, 23, 24), reducing disability and recovery (14, 18, 22, 24), improving quality of life (14, 18, 23, 24), reduced symptoms and signs of neuropathy (2), reduced knee arthritis stiffness and improved performance (15, 19, 22), reduced pigmentation of varicose veins (23), increased walking strength distance (18), improved range of motion, and clinical significance in knee arthritis (19).

## 4 Discussion

LT, a traditional therapeutic modality, has demonstrated efficacy in managing various conditions. These included hormonal and metabolic complications, cardiovascular problems, and inflammatory-based diseases (10, 11, 25). Leech saliva contains therapeutic peptides that promote healing by acting as analgesics, blood vessel relaxants, bacteriostatics, anti-inflammatory agents, clotting agents, and antiedematous substances (26, 27).

In the present systematic review, it is demonstrated that LT can improve the quality of life (14, 18, 23, 24) Additionally, all medical disorders examined overall benefits from LT, including knee osteoarthritis (15–22), epicondylitis (14), varicose veins (23), chronic back pain (24), and type 2 diabetics with neurogenic diseases (26). Except for one study on osteoarthritis of the knee (21), which failed to demonstrate any beneficial effects, this study applied five leeches, the intervention lasted 30 min, and it is the only one that utilized NaCl to violently remove the leeches from the bite site. Hence, it seems that the therapeutic advantages of this procedure are predominantly attributable to the leech’s spontaneous discharge and the duration of treatment.

Leech numbers used in the research ranged from 2 to 8. Although considering the procedure’s success with various numbers, it does not appear that leech numbers had an impact on the outcomes of this treatment method. However, applying a limited number of leeches does not seem reasonable for the probability that leeches may not bite. The number of leeches also increases the likelihood of being bitten. Of course, it should be mentioned that the frequent use of this therapeutic procedure or the high number of leeches can have an impact on blood elements. The research of M. Isik’s LT, which applied five leeches once a week for 3 weeks, reduced patients’ hemoglobin and hematocrit levels (16).

In this study, LT was effective for any duration. As mentioned, the minimum duration of use was 30 min, and the maximum duration was 70 min. In other studies, a period of 3,090 min has been suggested (28, 29). Sufficient evidence of the effect of the duration of LT on the treatment results was not found. On the other hand, it is suggested in various studies that the leech is automatically separated from the place (14, 15, 20, 28). Sometimes, it is necessary to remove the leech from the treatment site in a non-spontaneous manner. For example, when the leech does not increase in volume after 30 min and is inactive (29) or it leads to severe complications, in which case care must be taken not to enter the contents of the leech into the wound (30). Therefore, it seems that it is better to remove the leech from the place by itself, in which case the duration of the intervention does not have a significant difference in the effective treatment results.

The results of this study show that the use of LT is not associated with severe side effects, and according to Maetz et al.’s study, infection caused by LT cannot be ignored and is one of the important side effects of this treatment (31). In another study, it was found that among the studies conducted on humans, 79.05% used antibiotics after leech treatment (32). As stated in the present study, prolonged bleeding is one of the common side effects of leech use that must be managed (33). Management of possible complications makes LT a useful and complementary treatment method (5).

In general, the results of the present study show that the use of leech and its saliva can be useful in the treatment of many diseases. Alaama et al. also recommend the use of leech, especially for complex diseases such as cancer and diabetes (34). Another use of LT is in the problems of venous congestion and replantation (35).

In the present study, we only examined controlled interventional studies and did not consider other types of studies, such as non-randomized studies, which was due to resource limitations and high bias in reporting results in other studies.

## 5 Conclusion

LT serves as an effective treatment modality within traditional medicine for managing diverse conditions. While this approach may pose complications, it remains feasible to benefit from this cost-effective and minimally complex treatment method by implementing preventive measures.

## Data Availability

The original contributions presented in the study are included in the article/supplementary material, further inquiries can be directed to the corresponding author/s.
